# Speech disturbance plays critical role in stroke recognition during COVID‐19 pandemic

**DOI:** 10.1111/cns.13608

**Published:** 2021-01-16

**Authors:** Renyu Liu, Marc Fisher, Anthony Rudd, Jing Zhao

**Affiliations:** ^1^ Department of Anesthesiology and Critical Care Perelman School of Medicine at the University of Pennsylvania Philadelphia PA USA; ^2^ Department of Neurology Beth Israel Deaconess Medical Center Harvard Medical School Boston MA USA; ^3^ Stroke Medicine Kings College London London UK; ^4^ Department of Neurology Minhang Hospital Fudan University Shanghai China

The novel coronavirus disease pandemic (COVID‐19) has had a profound impact on stroke and stroke care. We and others have reported that there was a significant drop in stroke admissions and the numbers of cases treated with thrombolysis and thrombectomy across the world. National data registry in China showed that there was a 40% drop in stroke admission throughout China during the height of the pandemic.^1^ In the United States, a 39% drop in stroke evaluations was reported.^2^ From our international survey done through World Stroke Organization, we found that stroke admissions dropped on average 40% across the world.^3^ One contributory factor could have been due to the missed opportunities to recognize stroke during the pandemic. Due to social distancing and facial masking, facial drooping, one of the common signs of stroke, could potentially be covered and not easy to be recognized from a distance. Wearing a mask might also have made recognition of mild dysarthria more difficult. With social distancing, arm weakness, another common signs of stroke, may not easily recognized either. Therefore, many mild stroke patients might have been missed, and some of them may have then gone on to have a major stroke.

Human‐human interaction shifted dramatically from face‐to‐face interaction to a more virtual one after the onset of the COVID‐19 pandemic. Virtual communication including telephone communication, text messaging, and videoconferences became the mainstream communication and interaction methods. Many hospitals including some in rural areas have implemented stroke telemedicine networks equipped with video camera and medical providers who are trained to recognize stroke using FAST (Face, Arm, Speech, Time) tool.^4^ Speech disturbance, one of the critical signs of stroke, can usually be easily identified which should trigger an emergency medical service call (911 in USA, 999 in England, 112 in most of the EU countries, and 120 in China for example). Here are two typical examples that triggered massive press interest in the United States. Former presidential candidate Ron Paul suffered a stroke during a live interview. Reviewing the interview video, the first sign to appear is that he developed very slurred speech followed by drooping of the face with a crooked mouth.^5^ Another example is that a schoolteacher overheard the slurred speech of an elderly person in the background of the house of one of her students when the student was online for her class. The teacher acted immediately to arrange a call to 911, because she believed that the person who had slurred speech might be having a stroke. Her action was potentially life‐saving.^6^ Both stories highlight the usefulness and importance of recognizing speech disturbances even in a remote situation to identify stroke and allow for timely intervention.

To speak normally is a complicated process. It relies on many parts of the brain working cooperatively together, involving language comprehension, processing, and motor coordination of muscle groups governed by different parts of the motor cortex. Speech formation and comprehension are primarily controlled by the frontal and temporal lobes of the cerebrum; Broca's area, located in the frontal lobe of the dominant hemisphere of the cerebrum, is responsible for converting thoughts and ideas into spoken words; Wernicke's area, located in temporal lobe, is primarily responsible for understanding and processing speech. In addition to the motor cortex in the cerebrum, the cerebellum is also needed to coordinate specific muscle groups to move the mouth, tongue, and part of the face related to facial expression. The arcuate fasciculus allows for communication between these two important speech areas. Any damage to this complicated process will result in the various forms of speech disturbance, including either aphasia (difficulty in speaking or understanding) or dyspraxia/ dysarthria (difficulty in using muscle group for speaking). While the left brain controls language in right handed people and some left‐handed individuals in fact all people need both hemispheres for completely normal speech which includes an ability to show emotion in speech. Therefore, speech disturbance can be seen in both left‐ and right‐sided brain injury induced by stroke. In the early studies for the development of FAST, it was reported that 72% of the confirmed stroke patients by a physician had speech disturbance, 62% had facial drooping, and 87% had arm weakness.^7^, ^8^ Motor dysfunction or speech impairment is more reliable in predicting a stroke than facial drooping.^8^ A recent study indicated that speech disturbance has a strong predictive value for worse outcome after stroke compared with individuals where speech is unaffected.^9^, ^10^ The incidence of speech disturbance is as high as 84.4% in stroke patients, and a speech disturbance persists at discharge in 75.8% of patients who survived their stroke.^10^ Therefore, speech disturbance is not only a sensitive marker of stroke, but also has a predictive value for outcome. Aphasia can be used as an important tool to screen possible large vessel occlusion stroke.^11^ Potential large vessel occlusion recognition in the prehospital setting is critical, so the patient can be directly sent to stroke center that has thrombectomy capabilities to avoid delay due to retransfer. While the therapeutic window for thrombectomy has extended significantly, the principle of early intervention does not change for a favorable outcome.^12^, ^13^, ^14^

To emphasize the importance of the speech disturbance and connect the stroke sign and symptoms to the emergency number as we have proposed in the past for the Stroke 1–2–0 and Stroke 1–1–2 programs,^15^, ^16^ we, hereby, propose that the Stroke 9–1–1 (abbreviated as S911 or S‐9–1–1) program test whether such a program could potentially work better for stroke identification than FAST, especially during the pandemic where masking and social distancing are needed. As indicated in Figure 1, we set the speech disturbance as the first and most important sign to be recognized. We believe it is critical to have the name of “Stroke” in the educational program, and S stands for “Stroke” and “Speech Disturbance.” The direct linkage among the acronym, the stroke signs, and symptoms might help to trigger the initiation of the stroke rescue cascade by using the emergency medical system, rather than using other transportation methods. In this strategy, we moved speech disturbance and arm weakness ahead of an uneven face since the uneven face is less easily identified and less specific based on the incidence of the finding in confirmed stroke cases.

**FIGURE 1:**
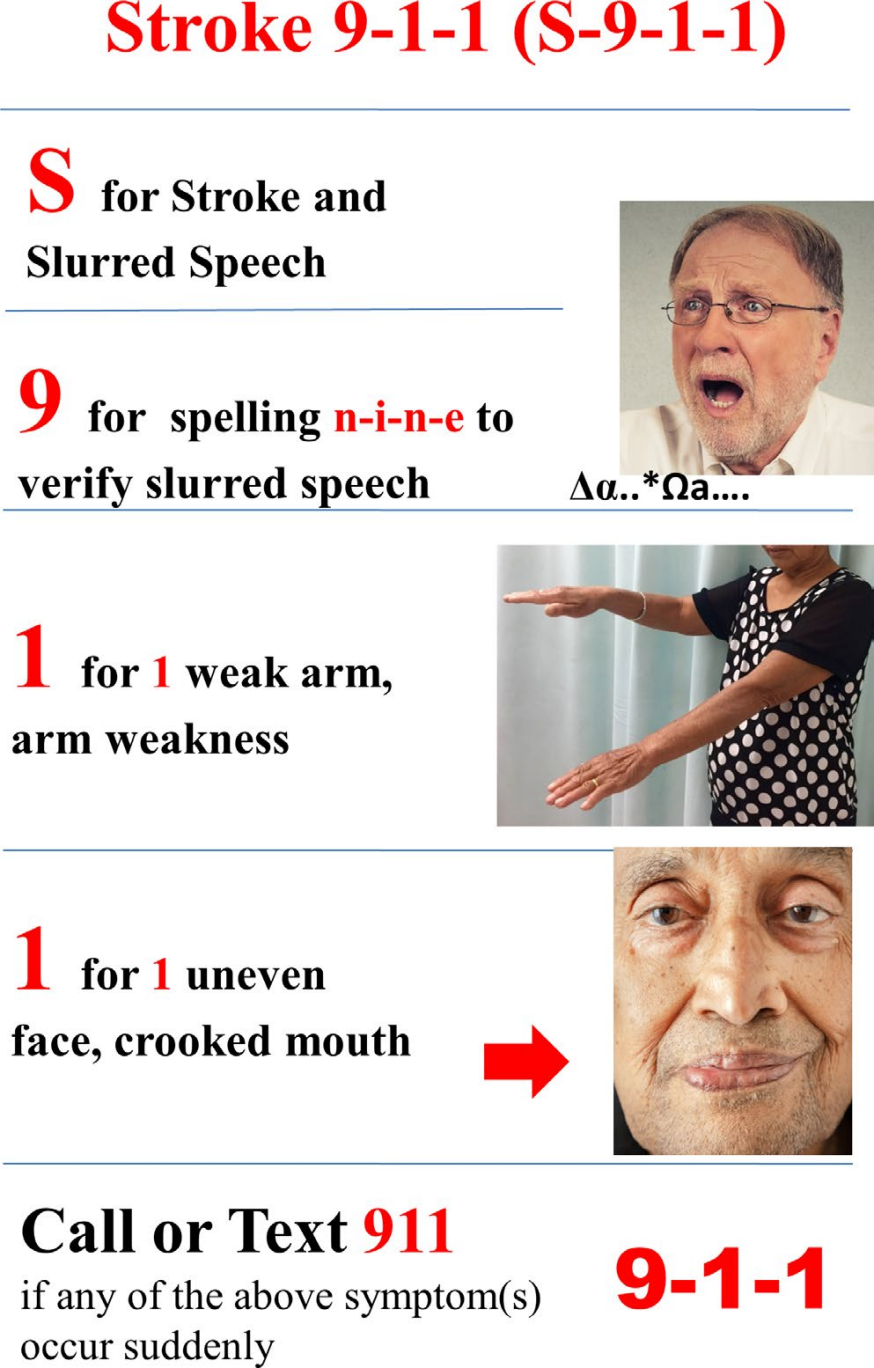
The proposed Stroke 9–1–1 public educational program. We have obtained copyrights for all the individual photographs used in this figure

## CONFLICT OF INTEREST

None.

## AUTHOR CONTRIBUTIONS

Dr. Liu proposed the concept, reviewed literature, and drafted and wrote the manuscript; Dr. Fisher and Dr. Rudd reviewed literature and wrote the manuscript; Dr. Zhao provided images and wrote the manuscript.

## Data Availability

Please contact Dr. Renyu Liu if you want the high resolution image of the figure for educational purposes. No other original data is used.
